# A bibliometric analysis of optic atrophy from 2003 to 2023: research trends and hot spots

**DOI:** 10.3389/fmed.2024.1497446

**Published:** 2025-01-06

**Authors:** Liyuan Wang, Tianyang Yu, Runze Wang, Lijuan Fu, Feixue Dong, Shuang Zhao, He Sun, Yang Gao

**Affiliations:** ^1^School of Clinical Medicine, Heilongjiang University of Chinese Medicine, Harbin, China; ^2^Department of Ophthalmology, First Affiliated Hospital of Heilongjiang University of Chinese Medicine, Harbin, China; ^3^Graduate School, Heilongjiang Academy of Traditional Chinese Medicine, Harbin, China; ^4^Department of Acupuncture, Second Affiliated Hospital of Heilongjiang University of Chinese Medicine, Harbin, China; ^5^Department of Rehabilitation Medicine, Qingdao Eighth People’s Hospital, Harbin, China; ^6^Department of Acupuncture, Qingdao Central Hospital, University of Health and Rehabilitation Sciences (Qingdao Central Hospital), Qingdao, China

**Keywords:** optic atrophy, bibliometric analysis, research trend, collaborative network, Web of Science

## Abstract

**Background:**

Optic atrophy (OA) is primarily caused by damage to the retinal pathway system, including widespread degeneration of retinal ganglion cells and axons, leading to visual impairment and blindness. Despite its clinical significance and diverse etiological factors, there is currently a lack of comprehensive bibliometric analyses exploring research trends and hotspots within this field.

**Method:**

This study retrieved relevant literature on OA published between 2003 and 2023 from the Web of Science Core Collection database. We conducted a bibliometric analysis using tools such as CiteSpace, VOSviewer, and SCImago Graphica to examine annual publication trends, co-occurrence patterns, collaborative networks among countries and institutions, and the evolution of research hotspots of OA.

**Results:**

A total of 5,274 publications were included in the bibliometric analysis, comprising 4,561 research articles and 713 review articles. The United States emerged as the leading country in OA research, followed by Germany and China. Over the past two decades, the primary research hotspots focused on “mitochondrial dysfunction,” “hereditary optic neuropathy,” “ocular hypertension” and “diagnostic techniques.” Future research trends are likely to revolve around “molecular mechanisms” and “therapeutic targets.”

**Conclusion:**

This bibliometric analysis provides an overview of research developments in OA over the past 20 years, highlighting the emphasis on the pathological basis of OA and advancements in diagnostic and therapeutic approaches. Future studies should continue to explore the molecular basis of mitochondrial dysfunction to identify potential gene therapy targets for treating OA.

## 1 Introduction

Optic atrophy (OA) is primarily caused by damage to the retinal pathway system, including the widespread degeneration of retinal neurons, ganglion cells, and axons (1), and is characterized by the nonspecific manifestation of optic disc pallor (2). OA represents the terminal stage of disease processes that result in visual impairment and blindness (3, 4). It is a pathological term rather than a specific diagnosis (4, 5), with common causes including trauma, vascular conditions, inflammation, tumors, and hereditary factors (4). Identifying the original cause of OA remains a priority, as it not only helps control the severity of the condition but also prevents the onset of potentially life-threatening diseases. Furthermore, understanding the molecular mechanisms is crucial for managing hereditary optic neuropathy.

Recent research has increasingly emphasized the role of systemic and genetic diseases in the progression of OA. For instance, diabetes has been shown to accelerate retinal ganglion cell degeneration, especially in glaucoma models independent of intraocular pressure (6). Conditions such as elevated intraocular pressure (IOP) and autoimmune diseases also contribute to optic nerve damage through altered blood flow or inflammation (7). Genetic factors are particularly significant, with hereditary optic neuropathies like Leber hereditary optic neuropathy (LHON) and dominant optic atrophy (DOA) linked to mutations in mitochondrial and nuclear genes, such as OPA1 (8–10). These genetic conditions lead to the progressive degeneration of retinal ganglion cells and axons, contributing significantly to OA. Over the years, various studies have provided valuable insights into OA treatment. Neuroprotective agents, such as caffeine, have emerged as promising options by reducing retinal inflammation (11). Additionally, NOX4 inhibitors are being explored for their potential to target oxidative stress, a critical factor in the pathophysiology of OA (12). Advances in drug delivery systems, such as lipid nanoparticles, offer new treatment avenues for OA (13).

Bibliometric analysis is a modern research methodology used to measure correlations within existing evidence, encompassing information about researchers, research institutions, publishers, and research content, thereby revealing the framework and evolution of a field (14). It highlights how research hotspots and trends shift over time and offers insights into the abundance, gaps, and trends of research within various subfields. This approach also helps identify biases and limitations in research across different periods (15). Despite the wealth of studies on OA, there remains a lack of comprehensive bibliometric analyses that track evolving research trends in this field. Moreover, there is limited understanding of scientific productivity in OA research, including the roles and interconnections among research institutes, researchers, and publishers. To address this gap, we conducted a bibliometric analysis of OA-related literature from the past 20 years using the Web of Science Core Collection (WoSCC) database and created a visual knowledge map to capture recent research trends, shape the modern understanding of OA, and provide a comprehensive overview of the field while identifying emerging research directions to guide future investigations.

## 2 Materials and methods

### 2.1 Data acquisition and search strategy

The publications for this study were sourced from the WoSCC, a highly respected global database well-suited for bibliometric and visualized analysis (16, 17). To minimize potential bias from database updates, all searches and data extractions were conducted on August 1, 2024. The study utilized the topic term “optic atrophy,” and the retrieval results were filtered using the following criteria:

Type of publication: only literature types categorized as “articles” and “review articles” were included.

Time frame: the publication date range for the retrieved literature was set from January 1, 2003, to December 31, 2023.

Language: only English-language publications were considered.

### 2.2 Data extraction and analysis

First, this study extracted general information from the search results using the analysis and citation reporting functions in the Web of Science (WoS). This included data on the number of publications and citations by year and country/region, as well as basic information about prolific authors, institutions, and journals. Microsoft Excel 2019 and Origin 2021 software were employed for basic visualization and summarization of this information. Secondly, we exported the literature as full records and references, saving them as plain text and tab-delimited files from WoS. These files were then used with CiteSpace (version 6.1.R3), VOSviewer (version 1.6.18), and SCImago Graphica (version 1.0.28) to perform a bibliometric analysis.

**Figure 1:**
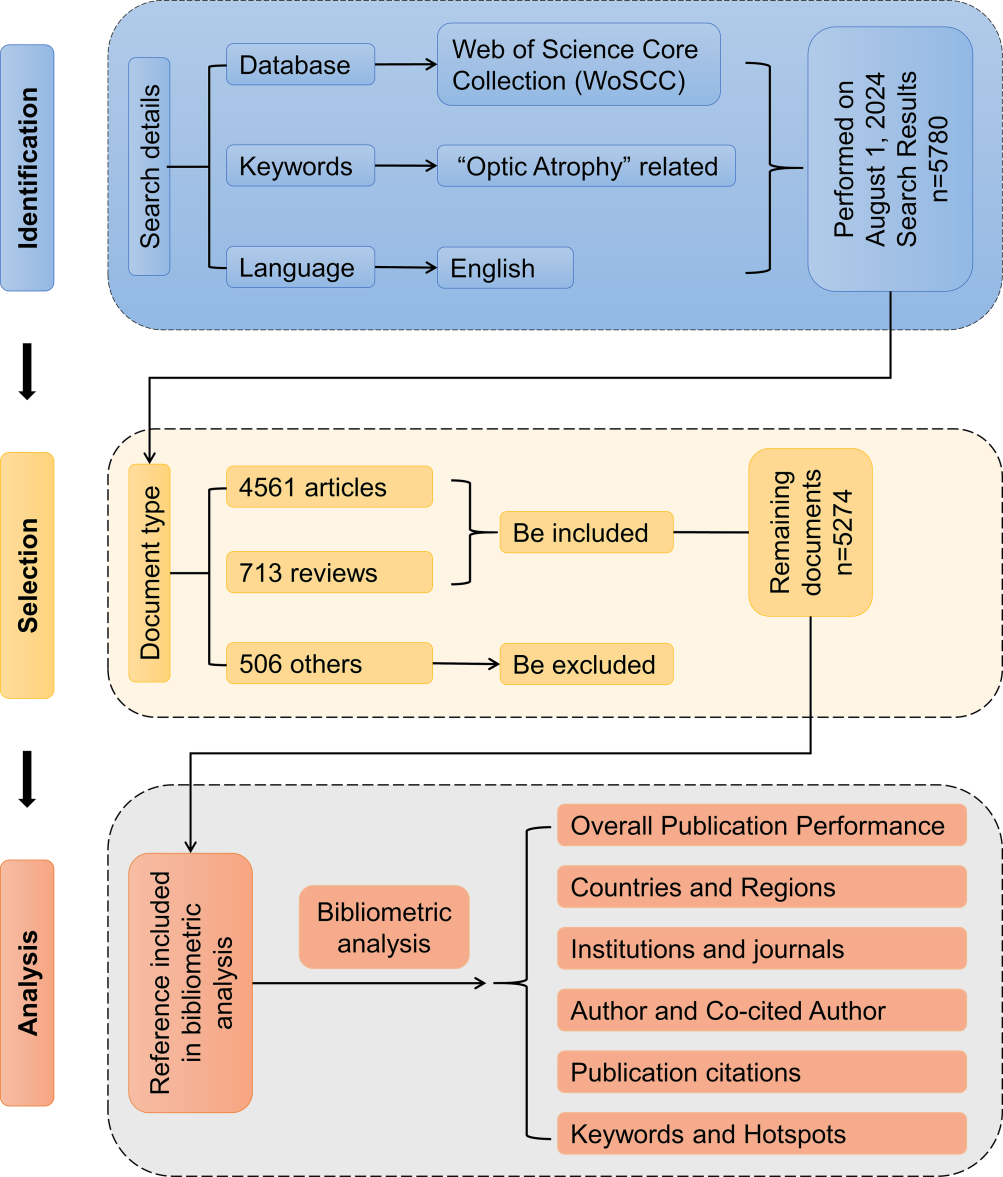
Flow chart of literature selection for bibliometric analysis.

The flowchart of the literature selection and bibliometric analysis was presented in Figure 1.

## 3 Result

### 3.1 Overall publication performance

**Figure 2:**
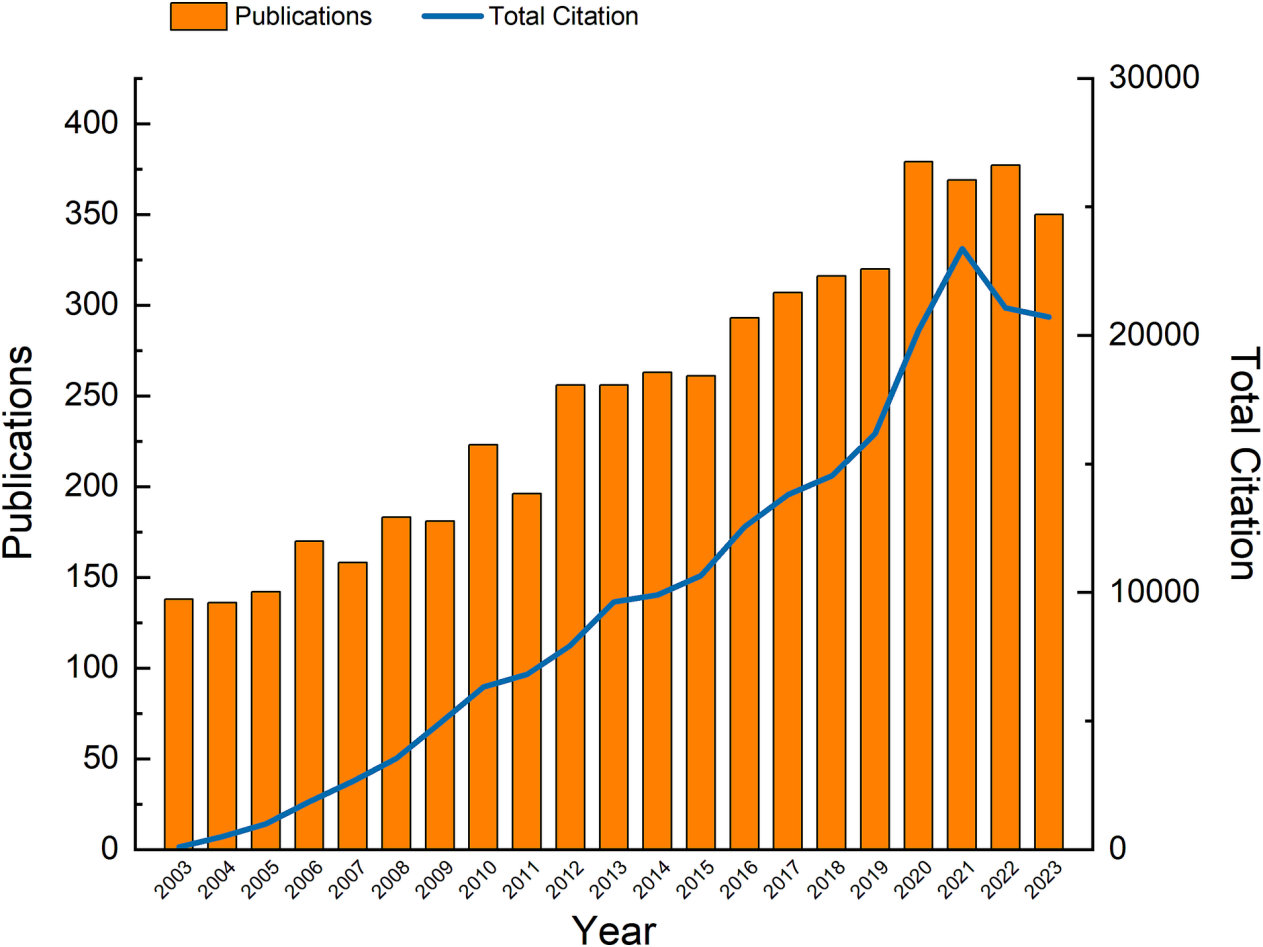
Yearly publication distribution and growth trend from 2003 to 2023.

We retrieved a total of 6,010 records from all related articles published between January 1, 2003, and December 31, 2023. After filtering for English-language publications and selecting only articles or review articles, we used both histograms and line graphs to display the number of articles published and the citations per year from 2003 to 2023, revealing research trends in OA. As shown in Figure 2, a total of 5,274 publications were included in the study over the past 20 years, comprising 4,561 articles and 713 review articles. The results indicated that there were 7 years (2004, 2007, 2009, 2011, 2015, 2021, 2023) in which the number of publications declined. Among the years with an increase, 2012 and 2020 exhibited significant growth, with increases of 30.6 and 18.4%, respectively. Meanwhile, the number of citations showed a clear upward trend until 2023, when a temporary decline was observed, likely due to citation lag for recently published articles.

Based on these data, research papers on OA have generally experienced a prosperous period of steady growth over the past 20 years. However, the declining trend in the number of publications and citations in the past 3 years may suggest that the field is entering a phase of stabilization or transition.

### 3.2 Countries and regions

**Figure 3:**
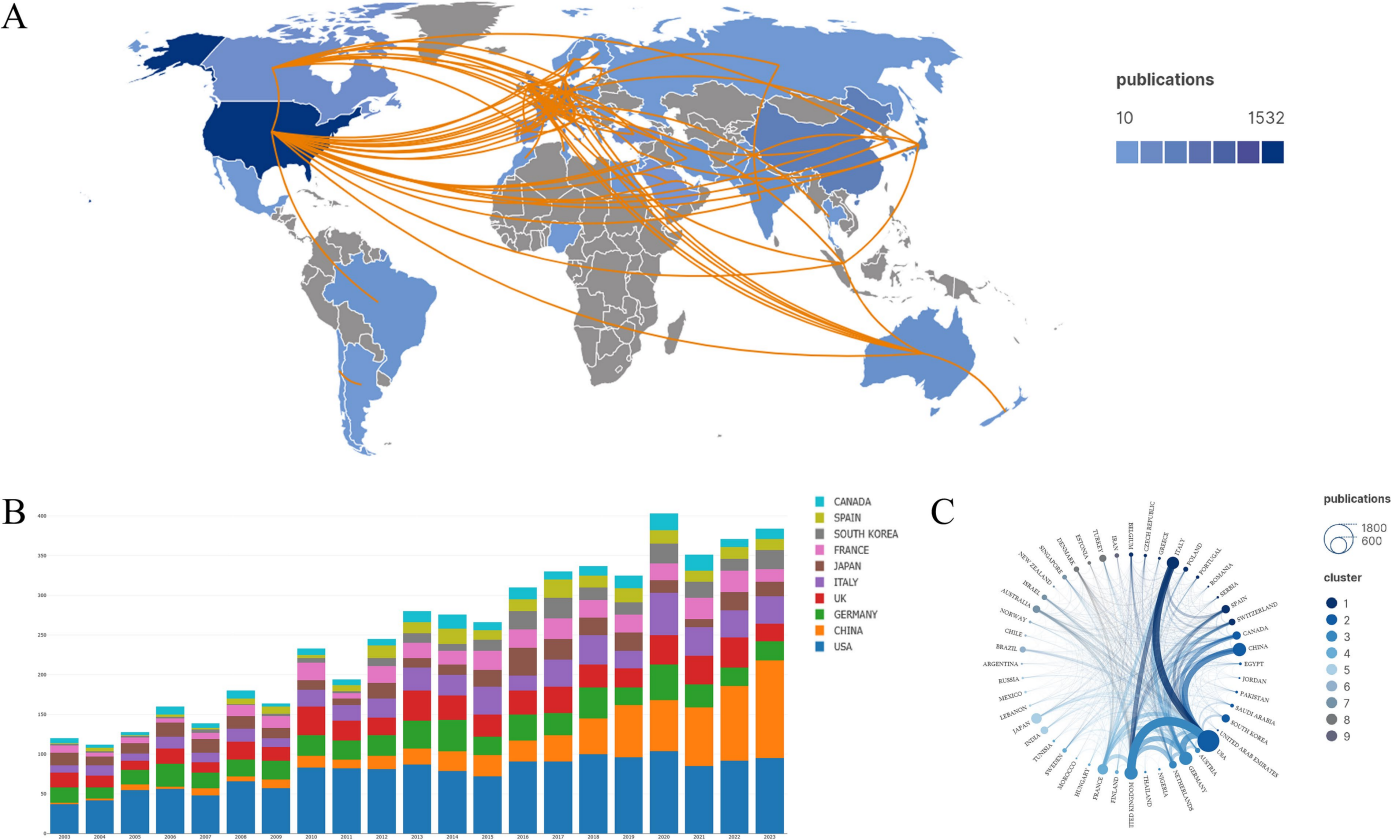
Countries and regions. **(A)** Geographical distribution and cooperation among countries related to OA publications. **(B)** Annual publication volume by each country. **(C)** Collaborative relationships between countries and regions.

The geographical distribution of global literature on OA (Figure 3A) reveals that a total of 48 countries have contributed to OA publications, with most of these countries located in North America, Europe, and Asia. In North America, the USA leads with 1,545 publications. In Europe, Germany, the UK, and Italy rank second, fourth, and fifth with 553, 504, and 486 publications, respectively. In Asia, China ranks third with 505 publications. These countries have collectively contributed over 70% of all literature in the field of OA over the past two decades. However, as shown in Figure 3A, there are still notable research gaps in many countries, suggesting potential opportunities for further development in these regions.

The stacked histogram of annual publication volumes by each country (Figure 3B) indicates that there has been no significant change in the volume of published literature over the past 20 years for the USA, UK, and Germany. In contrast, China shows a clear upward trend, suggesting that researchers in this region may be making new discoveries in OA research. Figure 3C illustrates international collaboration, highlighting that the USA is at the center of OA research, with the closest collaborations being with Italy, the UK, China, and Germany. Additionally, there is a high level of academic exchange between the UK and Italy in the field of OA. Overall, cooperation among countries in Europe and North America is strong, while collaboration with countries in other regions needs further strengthening.

### 3.3 Institutions

The top 15 institutions by the number of publications are listed in Table 1. The data extracted shows that over the past 20 years, 1,792 different organizations have been identified, with the top five institutions in terms of publications being the University of California System (252 publications), the University of London (250), Institut National De La Santé Et De La Recherche Médicale (204), Udice French Research Universities (202), and Centre National De La Recherche Scientifique (139). Total citations and the *H*-index reflect the level of interest and recognition of the literature by researchers in the field. The top five institutions based on total citations and *H*-index are the University of California System (15,381 citations/*H*-index of 65), the University of London (12,151/59), Institut National De La Santé Et De La Recherche Médicale (9,326/58), and Johns Hopkins University (11,019/52).

**Table 1:** Top 15 most productive institutions between 2003 to 2023 on OA.

Rank	Institution	Country	Number of documents	Total citations	Average citation	*H*-index
1	University of California System	USA	252	15,381	62.17	65
2	University of London	UK	250	12,151	49.77	59
3	Institut national de la santé et de la recherche médicale	France	204	9,326	29.03	58
4	Udice French Research Universities	France	202	8,869	45.2	57
5	Centre national de la recherche scientifique	France	139	6,287	47.48	44
6	Johns Hopkins University	USA	121	11,019	92.89	52
7	Moorfields Eye Hospital NHS Foundation Trust	UK	119	5,962	51.45	41
8	Ruprecht Karls University Heidelberg	Germany	116	4,436	39.76	38
9	Harvard University	USA	109	5,133	47.28	36
10	University of Bologna	Italy	108	6,071	60.81	45
11	Assistance publique Hôpitaux Paris	France	96	4,957	52.18	42
12	Helmholtz Association	Germany	86	4,794	56.66	37
13	Newcastle University UK	UK	85	6,942	83.92	42
14	Université Paris Cité	France	84	4,111	49.56	40
15	Université d'Angers	France	83	4,021	54.07	33

**Figure 4:**
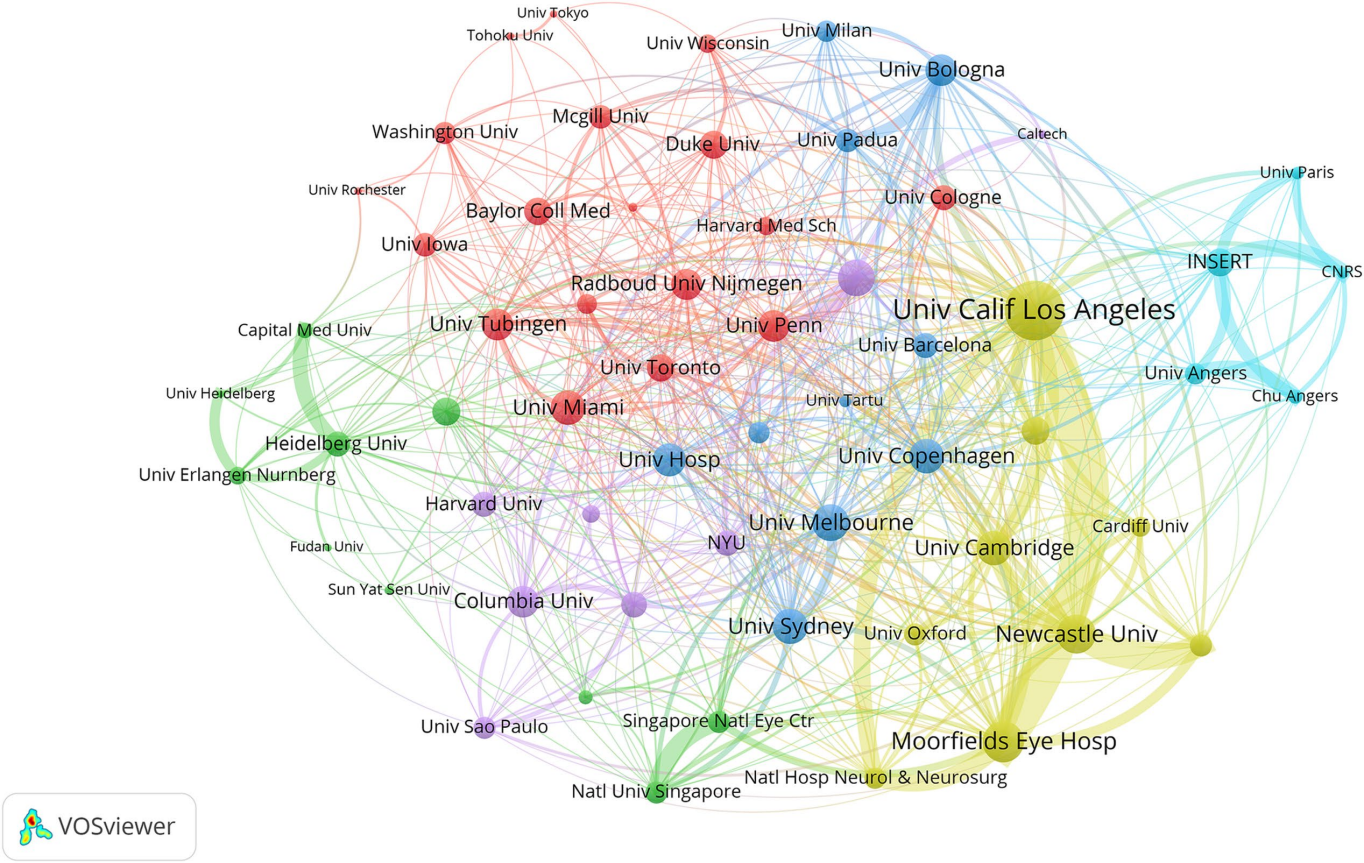
Co-occurrence analysis of major institutions involved in OA research. The thickness of the lines between institutions indicates the frequency of collaboration, with thicker lines representing more frequent cooperation.

To explore inter-institutional collaboration, we used VOSviewer to map the network of institutional collaboration and adjusted the scale’s weights to links to visually highlight the central institutions. As shown in Figure 4, the University of California System is the most central institution. Additionally, the figure indicates that institutions from the same country tend to cluster together, showing tight collaboration networks, while collaborations with institutions from other countries are less frequent. This pattern may suggest potential collaboration directions for future research development.

### 3.4 Author and co-cited author

**Figure 5:**
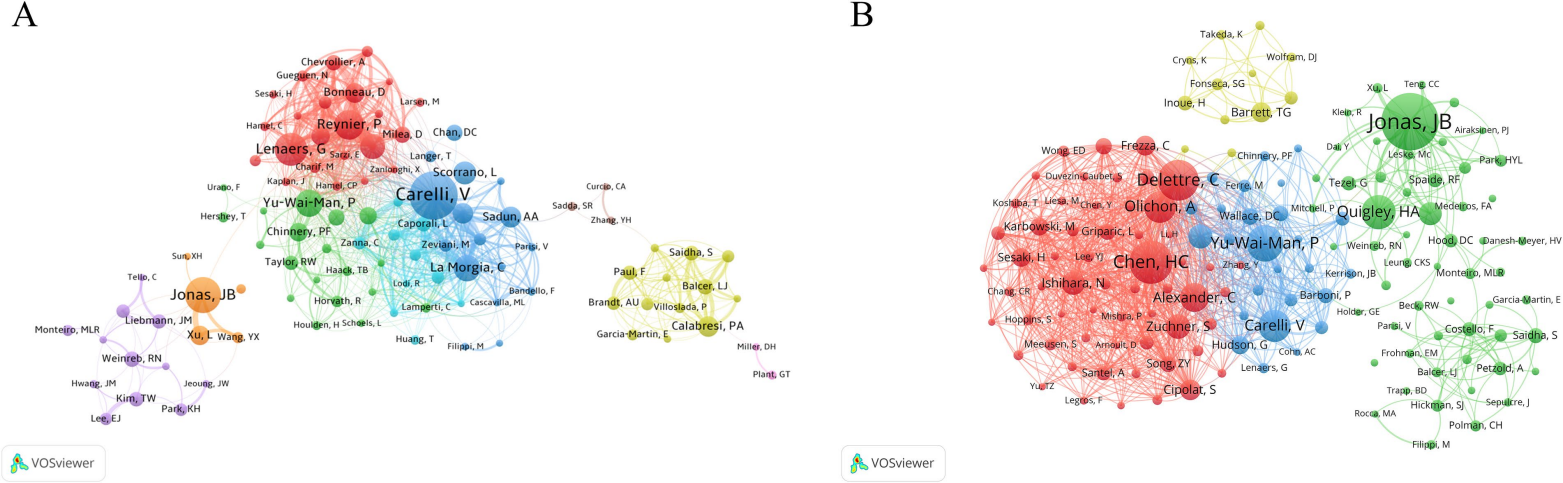
Author and co-cited author analysis. **(A)** Network analysis of authors involved in OA research. **(B)** Network analysis of co-cited authors in OA research. The thickness of the lines between authors indicates the frequency of collaboration, with thicker lines representing more frequent cooperation.

**Figure 6:**
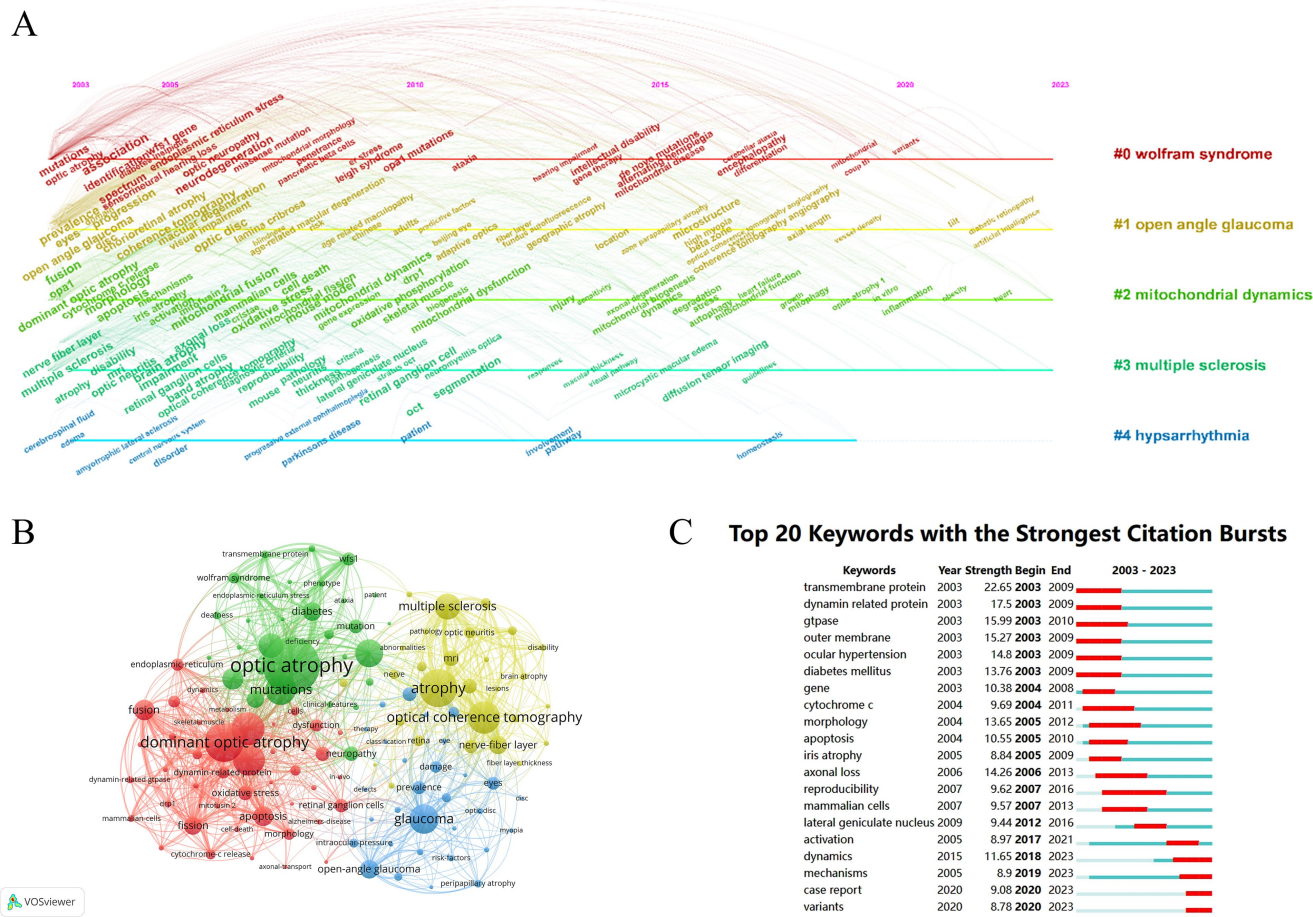
Keywords and hotspots. **(A)** A timeline view of the co-citation keywords appearance. **(B)** Visual analysis of keywords co-occurrence. **(C)** The 20 most cited keywords.

We retrieved the authors with the most publications related to OA from the WoS database and listed the top 15 most productive and most cited authors in Table 2. VOSviewer was used to map the network of co-authors and co-cited authors to reveal the collaboration patterns among researchers in OA, as well as to explore the frequency of citations and the relationships of “who cited whom.” Figures 5A,B display distinct clustering patterns. Among the most prolific authors, Carelli V. (91 publications) ranks first, Lenaers G. (59 publications) ranks third, Reynier P. (55 publications) ranks fourth, and Yu-Wai-Man P. (50 publications) ranks fifth. These authors are key connectors between research clusters related to mitochondrial dynamics (#2) as indicated on the timeline map of OA-related keyword in Figure 6A, demonstrating frequent collaboration. Jonas J. B. (76 publications), the second most prolific author, is more closely associated with cluster open-angle glaucoma (#1). The top five most cited authors over the last 20 years are Jonas J. B., Chen H. C., Delettre C., Yu-Wai-Man P., and Quigley H. A. This information suggests that prolific authors not only engage in closer collaborations but also significantly influence each other’s work.

**Table 2:** The top 15 authors in terms of number of publications and the top 15 co-cited authors in terms of number of citations on OA.

Rank	Author						Co-cited author	
	Name	Articles	Country	Total citations	Average citation	*H*-index	Name	Citations
1	Carelli V.	91	Italy	5,960	70.71	44	Jonas J. B.	1,362
2	Jonas J. B.	76	Germany	3,201	44.17	29	Chen H. C.	953
3	Lenaers G.	59	France	3,770	69.36	29	Delettre C.	887
4	Reynier P.	55	France	3,091	62.51	29	Yu-Wai-Man P.	770
5	Yu-Wai-Man P.	50	UK	3,426	71.86	25	Quigley H. A.	704
6	La Morgia C.	46	France	2,774	65.76	26	Carelli V.	650
7	Scorrano L.	40	Italy	6,694	171.28	30	Olichon A.	649
8	Bonneau D.	39	France	2,297	63.92	24	Alexander C.	572
9	Chinnery P. F.	38	UK	3,777	101.95	27	Zuchner S.	468
10	Votruba M.	37	UK	1,760	50.38	21	Amati-Bonneau P.	457
11	Calabresi P. A.	36	USA	3,519	101.44	24	Ishihara N.	435
12	Sadun A. A.	36	USA	2,648	76.08	25	Hayreh S. S.	432
13	Barboni P.	34	Italy	1,407	43.26	21	Cipolat S.	379
14	Procaccio V.	33	France	1,406	45.85	20	Barrett T. G.	358
15	Wissinger B.	32	Germany	1,619	53.66	20	Frezza C.	355

### 3.5 Journals

Table 3 lists the top 15 journals with the highest number of OA-related publications. The most frequently referenced journals are *Ophthalmology* (*n* = 8,275), *Brain* (*n* = 7,743), *Investigative Ophthalmology & Visual Science* (*n* = 7,709), *American Journal of Ophthalmology* (*n* = 3,247), and *PLoS One* (*n* = 3,213). According to the Journal Citation Reports 2021, 80% of the journals listed in Table 3 are ranked above Q2, indicating that OA-related articles have strong scientific impact and prestige. The journal with the highest impact factor is *Brain* (15.255), followed by *Ophthalmology* (14.277). These two journals are the third and eighth most prolific in terms of OA publications over the past 20 years, highlighting their active role in the field. Additionally, the *H*-index of these two journals ranks among the top three, underscoring that articles published in these journals are widely recognized and influential.

**Table 3:** The top 15 journals regarding the number of publications on OA.

Rank	Journal	Articles	Citations	Average citation	*H*-index	IF (2021)	QCR	5-year IF
1	Investigative Ophthalmology Visual Science	188	7,709	41.93	53	4.925	Q1	5.292
2	PLoS One	118	3,213	27.47	33	3.752	Q2	4.069
3	Ophthalmology	111	8,275	75.42	49	14.277	Q1	13.437
4	American Journal of Ophthalmology	98	3,247	33.5	32	5.488	Q1	6.048
5	British Journal of Ophthalmology	78	2,436	31.4	28	5.907	Q1	5.482
6	American Journal of Medical Genetics Part A	70	1,227	17.76	19	2.578	Q3	2.826
7	Graefes Archive for Clinical and Experimental Ophthalmology	70	947	13.6	18	3.535	Q2	3.394
8	Brain	67	7,743	116.84	50	15.255	Q1	16.173
9	Journal of Glaucoma	67	1,246	18.81	21	2.29	Q3	2.412
10	Scientific Reports	64	711	11.33	17	4.997	Q2	5.516
11	Journal of Neuro Ophthalmology	62	873	14.24	15	4.415	Q2	3.777
12	Eye	61	1,301	21.57	20	4.456	Q1	4.515
13	Acta Ophthalmologica	57	961	17.12	19	3.988	Q2	4.129
14	Ophthalmic Genetics	57	437	7.81	11	1.274	Q4	1.62
15	Journal of Neurology	49	1,444	29.76	22	6.682	Q1	6.174

**Figure 7:**
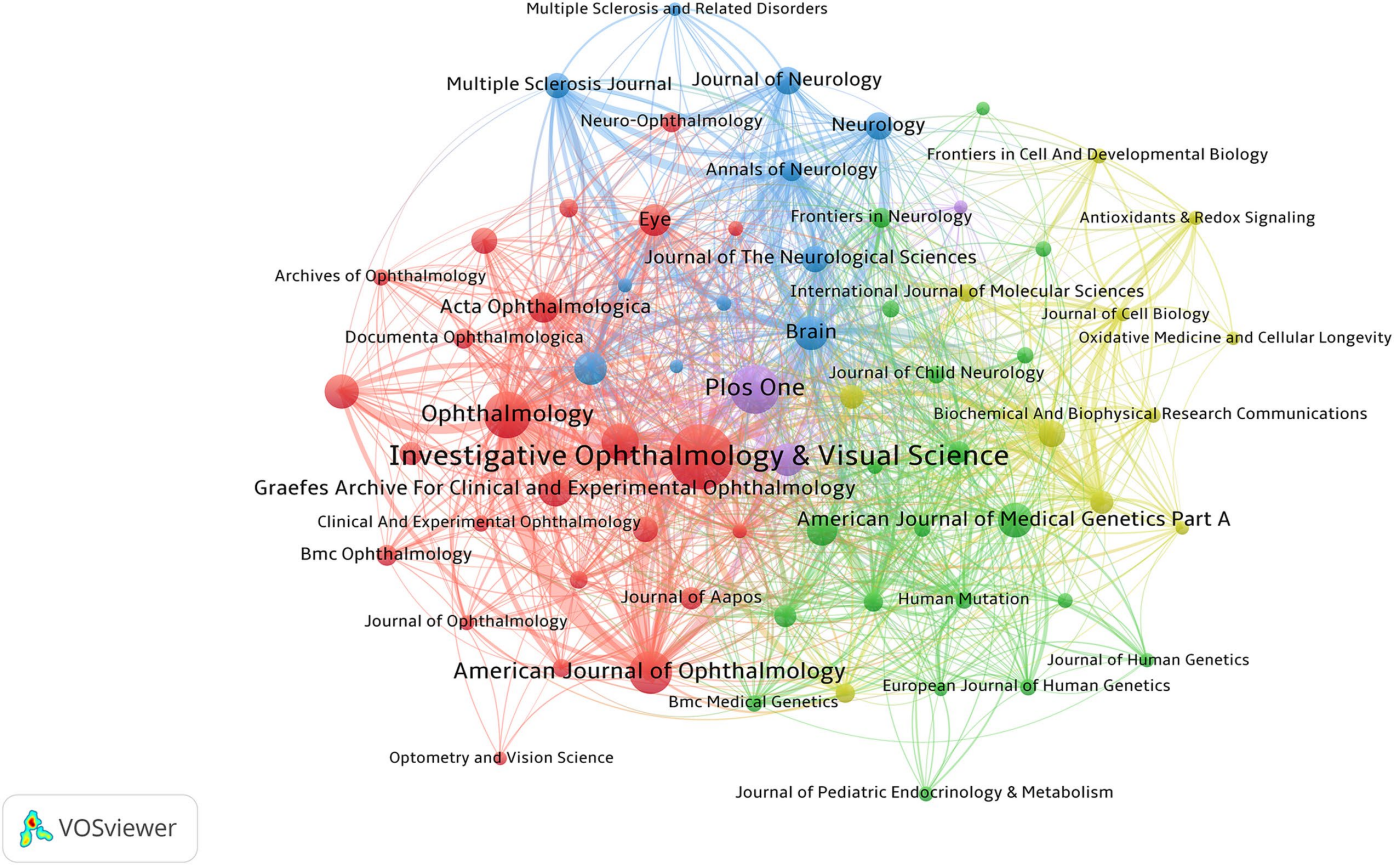
Co-occurrence analysis of the main journals involved in OA.

Figure 7 illustrates the research focus and collaboration network among different journals in the field of OA research. The figure reveals several distinct collaborative clusters, such as a neuroscience cluster centered around the *Journal of Neurology and Neurology*, and a genetics cluster focused on the *American Journal of Medical Genetics Part A*. This highlights the multidisciplinary nature of OA research and the close collaborative relationships between different fields.

### 3.6 Co-cited references

The top 10 most frequently cited references are listed in Table 4. The most co-cited paper is Chan’s (18) review, “*Mitochondrial dynamic organelles in disease, aging, and development*” published in *Cell* (*n* = 1,455). This is followed by Westermann’s (19) “*Mitochondrial fusion and fission in cell life and death*” in *Nature Reviews Molecular Cell Biology* (*n* = 1,302) and McBride’s et al. (20) “*Mitochondria: more than just a powerhouse*” in *Current Biology* (*n* = 1,295), which are the second and third most co-cited papers, respectively. The themes of these papers are predominantly related to neurological disorders caused by mitochondrial dysfunction, which has been a major focus and research hotspot in OA over the past 20 years.

**Table 4:** The top 10 most frequently cited references.

Rank	Title	Total citations	Publication year	Journal	First author	Type of paper
1	Mitochondria: dynamic organelles in disease, aging, and development	1,455	2006	Cell	Chan D. C.	Review
2	Mitochondrial fusion and fission in cell life and death	1,302	2010	Nature Reviews Molecular Cell Biology	Westermann B.	Review
3	Mitochondria: more than just a powerhouse	1,295	2006	Current Biology	McBride H. M.	Review
4	Mechanisms of disease: mitochondrial respiratory-chain diseases	1,157	2003	New England Journal of Medicine	DiMauro S.	Review
5	OPA1 controls apoptotic cristae remodeling independently from mitochondrial fusion	1,140	2006	Cell	Frezza C.	Article
6	The dynamin superfamily: universal membrane tubulation and fission molecules?	1,060	2004	Nature Reviews Molecular Cell Biology	Praefcke G. J. K.	Review
7	Mitochondrial dynamics-fusion, fission, movement, and mitophagy-in neurodegenerative diseases	1,027	2009	Human Molecular Genetics	Chen H. C.	Review
8	Functions and dysfunctions of mitochondrial dynamics	982	2007	Nature Reviews Molecular Cell Biology	Detmer S. A.	Review
9	Disruption of fusion results in mitochondrial heterogeneity and dysfunction	958	2005	Journal of Biological Chemistry	Chen H. C.	Article
10	Glycolytic oligodendrocytes maintain myelin and long-term axonal integrity	874	2012	Nature	Funfschilling U.	Article

**Figure 8:**
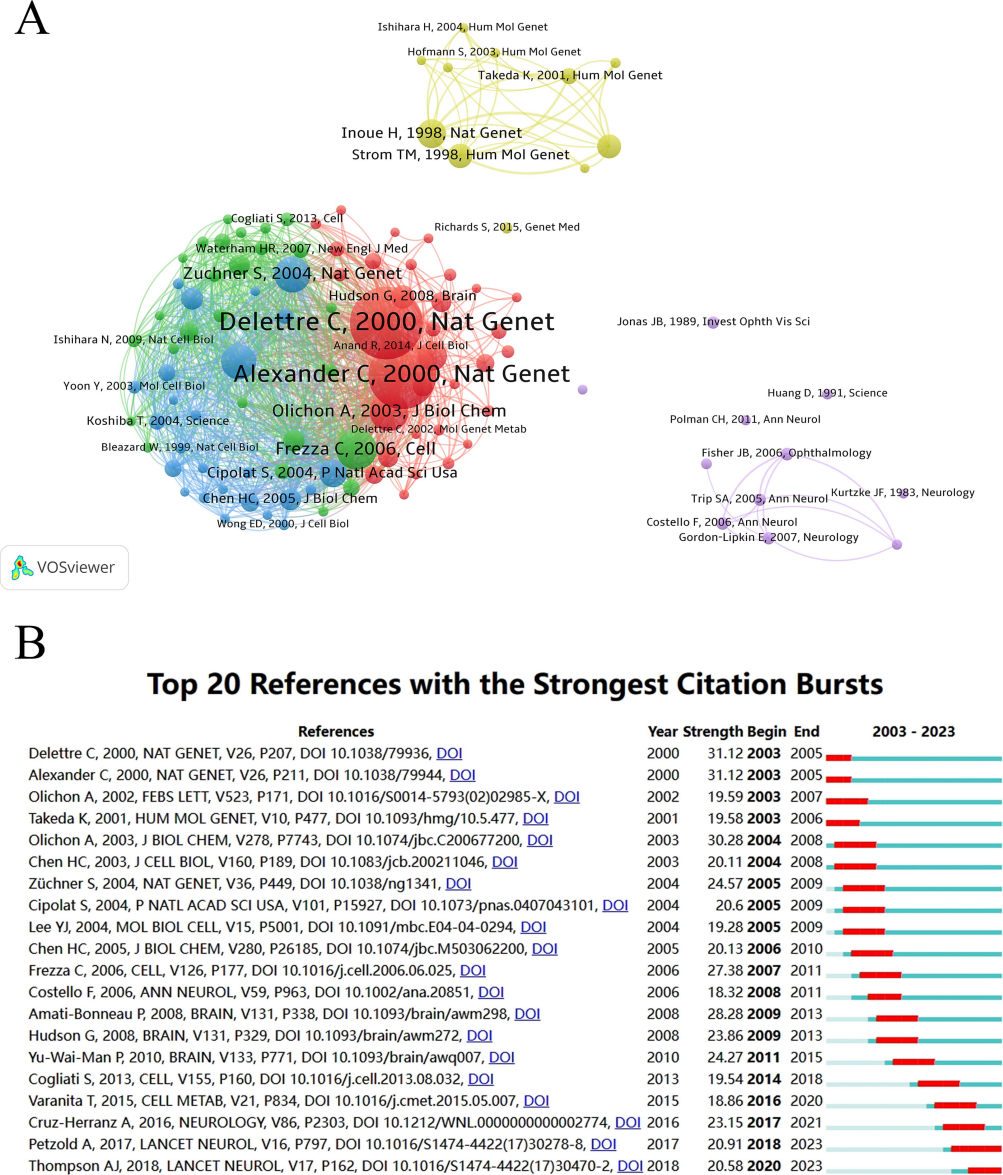
Co-cited references. **(A)** Network analysis of co-cited references in OA research. **(B)** Top references with the highest burst values in OA research from 2003 to 2023.

We identified references with more than 20 citations and used VOSviewer to plot the co-citation network. As shown in Figure 8A, references within the clusters related to mitochondrial dynamics, multiple sclerosis, and hereditary optic neuropathy are closely interconnected, demonstrating strong relationships and frequent co-citation among these topics.

Additionally, burst analysis results (Figure 8B) reveal that the three papers with the highest burst values are: “*The nuclear gene OPA1, encoding a mitochondrial dynamin-related protein, is mutated in dominant optic atrophy*” by Delettre et al. (21) (burst value = 31.12), “*OPA1, encoding a dynamin-related GTPase, is mutated in autosomal dominant optic atrophy linked to chromosome 3q28*” by Alexander et al. (22) (burst value = 31.12), and “*Loss of OPA1 perturbs the mitochondrial inner membrane structure and integrity, leading to cytochrome c release and apoptosis*” by Olichon et al. (23) (burst value = 30.28). All these articles were published between 2000 and 2003, collectively reveal how mutations in the OPA1 gene lead to mitochondrial dysfunction, which in turn causes optic nerve degeneration, thereby establishing a foundational framework for understanding one of the pathophysiology of OA. The burst values for the top 20 papers shown in Figure 8B are all above 18.32, highlighting the intense research interest in the OA field.

### 3.7 Keywords and hotspots

Research hotspots and trends can be identified by analyzing keywords in the literature. We plotted a timeline of the keyword network using CiteSpace (Figure 6A), which identified five clusters: “Wolfram syndrome,” “open-eye glaucoma,” “mitochondrial dynamics,” “multiple sclerosis,” and “hypsarrhythmia.”

We also used VOSviewer to map the correlation network of the keywords. To provide a clearer outline of the different clusters, we selected keywords with a frequency of occurrence greater than 50. As shown in Figure 6B, four clusters were identified between 2003 and 2023. Cluster 1 (green) is primarily related to the clinical manifestations and pathological mechanisms of Wolfram syndrome, such as deafness, diabetes, and the WFS1 gene. Cluster 2 (yellow) is associated with diagnostic tools like magnetic resonance imaging (MRI) and optical coherence tomography (OCT) in diseases where OA is a complication, such as multiple sclerosis, optic neuritis. Cluster 3 (red) focuses on the pathomechanisms of hereditary optic neuropathy, including keywords like dynamin-related protein, fusion, and fission. Cluster 4 (blue) pertains to OA resulting from glaucoma, with terms such as open-eye glaucoma, optic disc, and intraocular pressure.

Furthermore, the burst analysis results (Figure 6C) indicate that the five keywords with the highest burst values are “transmembrane protein” (*n* = 22.65), “dynamin-related protein” (*n* = 17.5), “GTPase” (*n* = 15.99), “ocular membrane” (*n* = 15.27), and “ocular hypertension” (*n* = 14.8). To highlight the most recent trends, we extracted keywords with the latest citation bursts starting within the last 5 years, including “activation,” “dynamics,” “mechanisms,” “case reports,” and “variants.” Notably, a total of 18 keywords have shown bursts for more than 5 years, demonstrating a continuous emergence of new research findings in the OA field over the past two decades.

## 4 Discussion

In this study, we employed various software and web platforms to analyze key trends in OA research, including the distribution of countries and regions, collaboration networks, and the evolution of research hotspots over the past 20 years. These tools are widely recognized in bibliometric studies for their ability to analyze and visualize large datasets. SCImago Graphica (24), a software designed for visualizing bibliometric data, which was used to explore citation patterns and relationships between countries. It provided insights into the global landscape of OA research and helped identify patterns of research collaboration and influence. VOSviewer specializes in constructing and visualizing networks based on relationships among authors, institutions, and research topics (25). This tool was applied to visualize networks involving institutions, authors, co-cited authors, journals, co-cited references, and keywords, which enabled us to identify key contributors and collaboration patterns within OA research. CiteSpace, a software tool designed to visualize trends and analyze scientific literature, was employed to track the evolution of OA research (26). It allowed us to perform a time-zone view analysis of OA-related keywords and conduct an exploded analysis of cited references and keywords separately, helping to identify emerging research concepts and cutting-edge developments. Additionally, CiteSpace generated clustered network diagrams, highlighting the key research topics and providing a clear view of how OA research has evolved over the past two decades.

Our analysis revealed that the publication of articles on OA has steadily increased since 2003, averaging more than 250 articles per year since 2012. However, it is noteworthy that, despite this steady growth, there does not appear to have been a significant breakthrough in the field of OA during the past two decades. The United States leads in OA research publications, with nearly three times the number of articles as Germany, which ranks second. Although China ranks third overall, it has shown a significant upward trend in publication growth over the 20-year period, surpassing both the UK and Italy. Before 2004, OA research in China was still in its early stages, characterized by substantial gaps. However, as awareness of OA increased and research funding expanded, China experienced a period of significant growth in OA research. Despite these developments, many countries still lack substantial research data on OA. This may be due to limited attention to this field in developing countries, insufficient research funding, a relative shortage of human resources, and lagging technological capabilities. Additionally, language barriers may contribute to incomplete data entries. Nonetheless, significant gaps in OA research are evident in certain regions and countries, underscoring the need for a more globally inclusive approach to OA research.

The University of California System is the most cited and most efficiently publishing institution in the field of OA research. Among the top 15 most prolific institutions, six are located in France, three in the USA, three in the UK, one in Italy, and one in Germany. As shown in Table 1, although China has seen an increase in publications and has strong international collaborations, it lacks a standout institution that consistently produces a large volume of OA research. This may be due to the presence of numerous research institutions in China with relatively balanced capabilities. The institutional collaboration network (Figure 4) highlights a close partnership between the University of California, San Francisco, and Moorfields Eye Hospital. Analyzing their co-authored articles reveals that their research primarily focuses on the diagnosis and clinical evaluation of conditions like glaucoma, multiple sclerosis, and other diseases that present with optic atrophy. One of the most cited papers from this collaboration is “*Consensus definition for atrophy associated with age-related macular degeneration on OCT: classification of atrophy report 3*” (27). This paper proposes an optical coherence tomography (OCT)-based definition for atrophy associated with age-related macular degeneration, classifying atrophy at different stages to promote consistent terminology in future studies. The second and third most cited papers are a meta-analysis of OCT in multiple sclerosis (MS) (28) and a cohort study examining the correlation between retinal thickness and the risk of MS progression using OCT technology (29). OCT is an imaging technique that rapidly generates cross-sectional images of the macula and parafoveal retina, allowing for quantitative analysis of retinal volume, thickness, and microvasculature in various regions (30). The use of OCT technology has significantly transformed the clinical evaluation and study of OA (31). The collaboration between the University of California, San Francisco, and Moorfields Eye Hospital has been instrumental in advancing the clinical application of OCT technology.

The authors’ analysis identifies Professor Valerio Carelli of the University of Bologna as the most productive author in the field of OA research. Carelli et al. (32) has proposed that increased production of reactive oxygen species (ROS) and chronic stress due to dysfunction in mitochondrial respiratory chain complex I are common characteristics of mitochondrial diseases. He also noted that retinal ganglion cells (RGCs) have high energy demands and are, therefore, more vulnerable to these related diseases. As a result, optic nerve atrophy is observed in many mitochondrial disorders, such as LHON, mitochondrial encephalomyopathy lactic acidosis stroke-like episodes (MELAS), and Leigh syndrome (33, 34). Mutations in mitochondrial DNA (mtDNA) are considered a causal factor in LHON. Carelli’s et al. (35) assessment of mtDNA in patients with LHON found that only a few patients shared the same haplotype, suggesting that most LHON mutations arise from independent factors. Furthermore, Carelli conducted a clinical study to evaluate the efficacy of gene therapy for LHON. He pooled data from a previously reported cohort of 208 external controls and compared the natural history of these patients with the best corrected visual acuity (BCVA) of 174 previously treated LHON patients. The study found that patients receiving rAAV2/2-ND4 injections were more likely to carry the same haploid mutation and had significantly higher BCVA in treated eyes compared to those with a natural disease course. The improvement in visual acuity was also more pronounced with bilateral gene therapy injections than with unilateral injections (36). Professor Carelli’s contributions have included significant insights into the role of mitochondrial dysfunction in the pathogenesis of LHON and the identification of corresponding gene therapy targets.

*Investigative Ophthalmology & Visual Science* has published the largest number of articles in the field of OA over the past 20 years and ranks first in *H*-index, indicating that its publications have received widespread recognition. Over 85% of the journals listed in Table 3 are ranked Q2 or higher, suggesting that OA articles are of high quality. This may be attributed to the diversity of diseases and the complex pathophysiological mechanisms that contribute to OA. The co-citation network diagram of journals reveals that, in addition to ophthalmology-related journals, OA research has also been published in neurology and genetics journals. This indicates that OA, as a clinical condition, is also relevant in the contexts of neurodegenerative and genetic disorders.

Among the top 10 most cited articles in OA research, eight are related to mitochondrial dysfunction. The co-citation network of references reveals a strong interconnection among studies focused on mitochondrial dynamics, neurodegenerative diseases, and genetic disorders, with these clusters significantly outnumbering others. This suggests that research on mitochondrial dysfunction in the context of neurodegenerative diseases and genetic disorders presenting with symptoms of OA is a major focus area in the field. The top three most cited articles in OA research have been highly influential in the broader field of mitochondrial research (18–20). Although these studies are not directly focused on OA, they are crucial for understanding mitochondrial dynamics, a key factor in OA pathogenesis. Specifically, mitochondrial dysfunction, including imbalances in mitochondrial fusion and fission, has been identified as a central mechanism driving the degeneration of retinal ganglion cells in conditions such as LHON and DOA (37). While these articles are more general in scope, they provide essential insights into the mitochondrial dysfunction that contributes to OA, particularly in terms of how mitochondrial damage leads to retinal ganglion cell degeneration. Their findings have significantly influenced OA research by laying the foundation for understanding mitochondrial dysfunction in optic nerve diseases.

Burst analysis identified the top three references with the strongest citation burst values, all of which were original articles. As shown in Figure 8B, these articles were published between 2000 and 2003 and have continued to receive sustained attention, underscoring their impact. Both Delettre et al. (21) and Alexander et al. (22) proposed that dominant inheritance of DOA could be caused by haploinsufficiency of the OPA1 gene, identifying four mutations—including missense, nonsense, deletions, and insertions—through genetic screening of families with dominant optic atrophy. Subsequently, Olichon et al. (23) further investigated the underlying pathological mechanisms and discovered that OPA1 deficiency disrupts the structure and integrity of the inner mitochondrial membrane, leading to cytochrome c release and apoptosis. Focusing on the past 5 years, the top four articles in terms of burst value are: Cruz-Herranz’s et al. (38) proposal for standardizing quantitative optical coherence tomography (OCT) studies, Petzold’s et al. (28) meta-analysis of retinal layer segmentation in multiple sclerosis, Varanita’s et al. (39) work on the role of OPA1 in controlling apoptosis and ischemic tissue damage through mitochondrial cristae remodeling, and Thompson’s et al. (40) revision of multiple sclerosis diagnostic criteria. These recent findings indicate that diagnostic techniques such as OCT have garnered substantial attention in OA research, demonstrating significant potential for further development. Additionally, mitochondrial dysfunction, particularly involving the OPA1 gene and mitochondrial cristae remodeling, has been explored in more depth in recent years. MS as one of the causes of OA, has also attracted increased focus, with a strong emphasis on its diagnosis, prognosis, and treatment (41).

The co-occurrence of keywords can reveal the distribution of research hotspots within a field, while burst analysis and keyword timelines offer insights into the evolution of research trends, helping to identify potential future directions. In the case of OA, as a pathological term, keyword clustering tends to center around the etiological factors leading to OA. As shown in Figure 6B, clusters 1 and 3 feature keywords indicating a focus on the genomics and molecular biology underlying the pathology, whereas clusters 2 and 4 highlight research on the clinical symptoms, diagnosis, and prognosis of the disease. This pattern may be due to clusters 1 and 3 being associated with genetic disorders such as Wolfram syndrome and DOA, respectively, while clusters 2 and 4 are centered around MS and glaucoma.

To further analyze the shifts in research hotspots in the field of OA, we plotted a keyword timeline and conducted a burst analysis. The timeline (Figure 6A) displays five clusters, with “mitochondrial dynamics” listed separately from the others, unlike in Figure 6B. The Wolfram syndrome cluster is highly relevant to OA research, as Wolfram syndrome, characterized by optic atrophy along with diabetes and deafness, shares a mitochondrial dysfunction pathogenesis that contributes to retinal ganglion cell degeneration, enhancing our understanding of OA in genetic disorders (42). Similarly, the open-angle glaucoma cluster highlights the significant overlap between glaucoma and OA, with elevated intraocular pressure causing optic nerve damage and retinal ganglion cell degeneration. This connection underscores the role of intraocular pressure and optic disc changes in OA pathogenesis (43). The mitochondrial dynamics cluster is central to OA, as mitochondrial dysfunction is a key feature in both hereditary and acquired forms of optic atrophy. Research in this area focuses on the role of mitochondrial fusion and fission in maintaining retinal ganglion cells, with mitochondrial dysfunction increasingly recognized as a critical factor in OA (32–34). The MS cluster highlights the link between neuroinflammation and OA, as optic neuritis in MS leads to significant optic nerve damage and atrophy. This research underscores the importance of neuroinflammation in retinal ganglion cell degeneration, a primary cause of OA in MS patients (40, 41). Lastly, the hypsarrhythmia cluster, although less directly related to OA, reflects the broader intersection of neurological disorders and ocular health, with rare cases of optic atrophy associated with seizure disorders (44). Although Wolfram syndrome, LHON, and DOA can all present with hereditary optic nerve atrophy, they do not share the same pathogenesis. Wolfram syndrome is an autosomal recessive disorder caused by endoplasmic reticulum dysfunction due to mutations in the WFS1 or WFS2 genes (42), whereas both DOA and LHON are associated with mitochondrial dysfunction (45). Since OA caused by mitochondrial diseases shares a common pathological basis, understanding the molecular mechanisms of mitochondrial dysfunction has become a crucial challenge in developing treatments for these conditions. We observe that the #2 keyword in Figure 6A spans a long duration, indicating that research on this topic has been a central focus in the OA field for an extended period.

Keyword burst analysis provides further insights. The top burst values keywords include “transmembrane protein,” “dynamin-related protein,” “GTPase,” “ocular membrane,” and “ocular hypertension.” These terms are closely linked to the molecular mechanisms of OA. Transmembrane proteins are essential for cellular integrity, and their dysfunction can lead to RGC degeneration (46). Dynamin-related proteins and GTPases play a critical role in mitochondrial dynamics, where their dysfunction contributes to mitochondrial fragmentation and OA (22, 47). Ocular membrane relates to the blood-ocular barrier, and its disruption, particularly in glaucoma, has been linked to optic nerve damage (48). Ocular hypertension, a key risk factor for glaucoma, has become more recognized for its contribution to mechanical damage of the optic nerve (43). These findings highlight the increasing focus on understanding the molecular and clinical aspects of OA, particularly through the lens of mitochondrial dysfunction. In the past 5 years, new keywords have emerged with high burst values, reflecting the evolving focus of OA research. Keywords like “activation,” “dynamics,” “mechanisms,” “case report,” and “variants” highlight key areas of current interest. Activation and dynamics reflect the exploration of molecular pathways and cellular mechanisms involved in OA, particularly in mitochondrial dysfunction. Mechanisms indicates an interest in the detailed biological processes driving disease progression. The term “case report” emphasizes the growing importance of clinical documentation and its role in advancing OA diagnosis and treatment (49). Lastly, the keyword variants reflect increased research on genetic mutations, particularly in OPA1, which is pivotal in hereditary optic neuropathies (21, 50). These recent keyword bursts suggest a shift towards a more integrated approach to OA research, combining genetic, molecular, and clinical perspectives.

## 5 Conclusion

Overall, after analyzing 5,274 articles, we found that the United States leads in terms of institutions, authors, and the overall number of publications and citations, underscoring its significant influence in the field of OA. The co-citation analysis over the past 20 years revealed that research on OA has primarily focused on the pathological basis of hereditary optic neuropathies and the diagnosis and treatment of MS-related OA. However, keyword burst analysis suggests that there is still a need for ongoing investigation into the molecular basis of mitochondrial dysfunction in the context of OA, with a focus on identifying suitable gene targets for the treatment of hereditary optic neuropathies such as LHON and DOA.

## Data Availability

The raw data supporting the conclusions of this article will be made available by the authors, without undue reservation.
